# Unlocking Microbial Dark Matter: A Comprehensive Review of Isolation Technologies from Traditional Culturing to Single-Cell Technologies

**DOI:** 10.3390/microorganisms14040933

**Published:** 2026-04-21

**Authors:** Xi Sun, Xiaoxuan Zhang, Jia Zhang

**Affiliations:** 1 College of Food Science and Bioengineering, Tianjin Agricultural University, Tianjin 300384, China; sunxi@tjau.edu.cn (X.S.); xiaoxuan20230707@163.com (X.Z.); 2 Tianjin Engineering Research Center of Agricultural Products Processing, Tianjin Agricultural University, Tianjin 300384, China; 3 Key Laboratory of Photoelectric Conversion and Utilization of Solar Energy, Qingdao New Energy Shandong Laboratory, Qingdao Institute of Bioenergy and Bioprocess Technology, Chinese Academy of Sciences, Qingdao 266101, China; 4 Shandong Energy Institute, Qingdao 266101, China; 5 University of Chinese Academy of Sciences, Beijing 101408, China

**Keywords:** uncultured microorganisms, microbial culture, traditional culture methods, single-cell technologies

## Abstract

Microorganisms represent the Earth’s most abundant biomass and a vast reservoir of genetic diversity. However, traditional agar plate methods fail to recover the vast majority of these species, leaving a “microbial dark matter” that holds immense potential for the discovery of novel antibiotics and bioactive compounds. While conventional techniques such as selective media and enrichment culture remain foundational, they are inherently limited by community biases and the inability to support low-abundance, oligotrophic species. To address these bottlenecks, a diverse array of innovative isolation strategies has emerged. This review systematically categorizes and evaluates these methodologies, ranging from in situ cultivation to high-resolution single-cell manipulation. We first examine membrane diffusion-based cultivation (e.g., iChip), which mimics natural microenvironments to resuscitate recalcitrant microbes. Subsequently, we explore high-throughput single-cell technologies, including microfluidics for physicochemical separation, optical tweezers for precise manipulation, and fluorescence-activated cell sorting (FACS). Special attention is given to Raman-activated cell sorting (RACS) as a label-free functional screening tool and reverse genomics for targeted capture. By synthesizing the strengths and limitations of these approaches, we propose integrated workflows designed to accelerate the mining of untapped microbial resources.

## 1. Introduction

Microorganisms constitute the most abundant and biologically diverse life forms on Earth. Ubiquitous in nature, they inhabit niches ranging from the human microbiome to extreme environments such as deep-sea hydrothermal vents, playing a pivotal role in global biogeochemical cycles and ecosystem stability. Despite more than a century of microbiological research, pure cultures obtained via traditional agar plate methods represent only approximately 0.1% to 1.0% of total microbial diversity. Consequently, the vast majority of microorganisms remain “uncultured” or “refractory to culture” often referred to as microbial “dark matter” [1,2].

Multiple factors make it difficult to achieve pure culture of these microorganisms under laboratory conditions. Biochemically, many microorganisms have complex and insufficiently understood nutritional and cofactor requirements. They thrive within narrow ranges of osmotic pressure, pH, and trace elements [3]. Some microorganisms grow extremely slowly or enter a viable but non-culturable (VBNC) state under environmental stress, making standard laboratory cultivation challenging [4]. Ecologically, many microorganisms do not exist independently. Rather, they depend on metabolic interdependency, spatial co-localization, co-evolution, and signaling interactions with other microbes or hosts to survive. Such finely tuned community structures and stable conditions are hard to replicate in a lab, resulting in poor growth when separated from their natural environment [5,6]. From an evolutionary and genomic perspective, some microorganisms residing long-term in nutrient-rich or specific ecological niches have undergone various degrees of genomic reduction. They have lost some self-synthetic metabolic pathways and stress response genes, relying more on external metabolic products and stable environmental conditions [7]. Although this evolution enhances resource efficiency within specific niches, it weakens their ability to adapt to nutritional variations and environmental fluctuations in artificial cultivation, increasing the difficulty of pure culture.

Concurrently, the escalating crisis of antimicrobial resistance has rendered many current anti-infective therapies insufficient for clinical needs. Furthermore, the discovery rate of novel lead compounds from traditionally culturable microbes has significantly dwindled. Driven by these dual challenges, there is an urgent imperative to systematically explore and exploit uncultured microbial resources, which remain the primary reservoir for novel antibiotics [8] and bioactive natural products [9,10,11,12]. This review systematically examines the evolution of isolation strategies, ranging from conventional plate culture-based techniques to emerging single-cell technologies. By analyzing these two primary dimensions, we provide a comprehensive overview of the current methodologies available for microbial strain isolation and cultivation.

## 2. Conventional Culture-Dependent Strategies: The Foundation and Its Limits

Traditional isolation techniques, primarily relying on agar plates and liquid broths, have served as the bedrock of microbiology for over a century. While these methods are indispensable for obtaining pure cultures (a prerequisite for physiological characterization and reference strain deposition) they are fundamentally limited by the “Great Plate Count Anomaly”. We categorize these foundational approaches into four primary strategies based on their selection mechanisms: dilution-based isolation, selective pressure, community enrichment and streaking purification for final clonal isolation. These four isolation methods are not independent. They work as basic technical modules that can be combined. In practical isolation workflows, researchers do not use only one method to complete pure culture. Instead, they combine them in flexible ways according to the sample type, the target group and the cultivation goal.

### 2.1. Dilution and Plating: The Gold Standard for Colony Isolation

**Figure 1:**
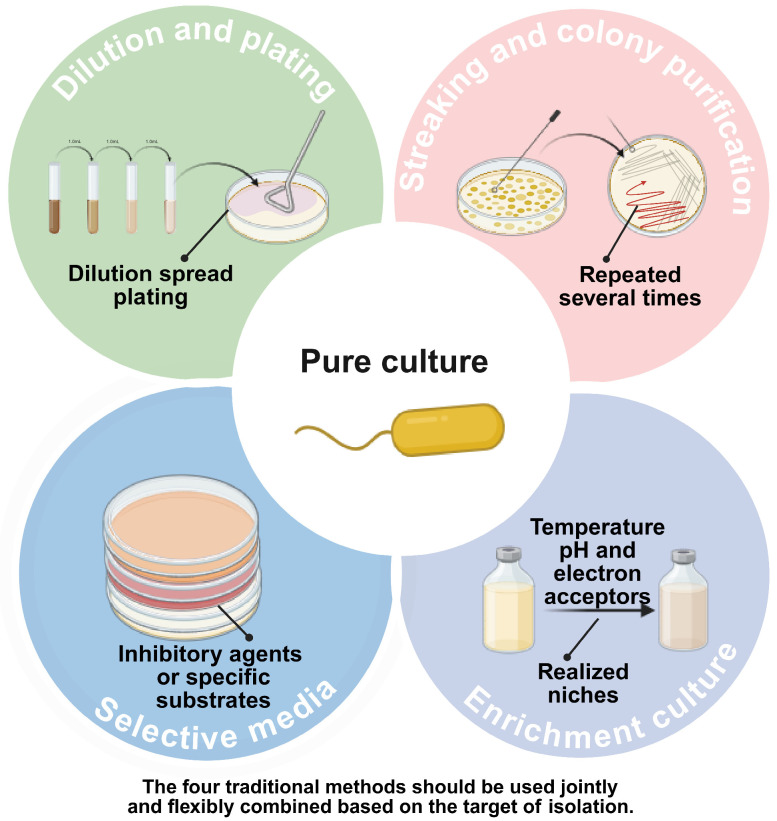
Schematic overview of traditional microbial isolation and cultivation strategies. The diagram illustrates the synergistic integration of four core approaches to achieve a pure culture: Dilution and plating for physical separation; Selective media utilizing inhibitory agents or specific substrates; Enrichment culture by manipulating environmental factors (e.g., temperature, pH) to simulate a realized niche; Streaking purification for final clonal isolation.

The spread plate method remains the most ubiquitous technique for separating microorganisms into discrete colony-forming units (CFUs). By spatially separating cells through serial dilution, this method facilitates the isolation of pure genetic lineages [13,14,15] (Figure 1). Its versatility allows for integration with modern molecular tools; for instance, Xun et al. combined plating with DNA sequencing to identify nectar-inhabiting strains [16]. Optimization of operational parameters, such as spreading duration, can further enhance recovery rates [17].

Despite its universality, the spread plate method suffers from significant ecological biases. It inherently favors “r-strategists” (fast-growing microorganisms that thrive in nutrient-rich environments) while failing to support oligotrophic “k-strategists” or those requiring specific symbiotic signals. Furthermore, the method is sensitive to initial cell density; low-abundance species are statistically unlikely to be isolated without prior enrichment. Consequently, this approach captures only a minute fraction of the microbial diversity present in complex environmental samples.

### 2.2. Selective Media: Targeting Traits via Nutritional and Environmental Pressures

Selective media are engineered to screen for specific microbial groups by exploiting their unique nutritional requirements or resistance profiles. This strategy functions by introducing inhibitory agents (e.g., antibiotics, salts) or specific substrates that permit the growth of target organisms while suppressing background flora (Figure 1). Applications range from isolating *Termitomyces* fungi [18] and slime molds [19] to detecting pathogens like *Listeria monocytogenes* using specialized formulations such as ASLM (Al-Zoreky-Sandine Listeria Medium) [20]. Recent advances include the CTC medium for entomopathogenic fungi [21] and antibiotic-supplemented media for Bifidobacterium [22].

The primary strength of selective media lies in their high specificity, making them the “gold standard” for pathogen detection and functional screening. However, the very mechanism of selection (stress) can be counterproductive. Inhibitory agents may suppress the growth of target strains that are sublethally injured or in a viable but non-culturable (VBNC) state. Moreover, this method requires a priori knowledge of the target’s physiology (e.g., antibiotic resistance), limiting its utility in discovering novel, uncharacterized taxa. It effectively filters known organisms but rarely uncovers the “unknown”.

### 2.3. Enrichment Culture: Amplifying Rare Species Through Ecological Selection

Enrichment culture operates on the principle of “realized niche” manipulation (Figure 1). By adjusting environmental parameters (such as nutrient sources, light, pH, or electron acceptors) researchers can directionally increase the relative abundance of specific functional groups within a mixed community [23]. This method has proven effective across diverse ecosystems: isolating biphenyl-degrading bacteria from polluted soil [24], analyzing prokaryotes in oil fields [25], and enriching polyhydroxyalkanoate (PHA) producers in wastewater [26]. Environmental cues are critical; for example, specific light wavelengths have been shown to dictate bacterial growth dynamics in deep-sea vent sediments [27], while low hydrogen concentrations favor specific methanogens [28]. Pre-enrichment strategies have also been demonstrated to expand the diversity of recoverable species from marine sediments [29,30] and aid in isolating extremophiles, such as arsenic-resistant *Acidithiobacillus* [31].

Enrichment culture is a powerful tool for bringing low-abundance (rare biosphere) species to detectable levels. However, it introduces severe community bias. The artificial conditions often select for the fastest-growing organisms (weeds) capable of utilizing the provided substrate, rather than the most ecologically relevant or efficient ones. This “bottle effect” can drastically skew community structure, potentially leading to the loss of slow-growing but functionally critical interactions. Furthermore, enrichment yields a mixed consortium, necessitating subsequent purification steps to obtain isolates. While enrichment successfully amplifies target populations, the resulting consortia require subsequent plating and purification (Section 2.4) to resolve individual strains.

### 2.4. Streaking and Colony Purification: Securing Monoclonality

Following initial isolation via dilution plating (Section 2.1) or enrichment (Section 2.3), subsequent purification is often required to eliminate residual contaminants and ensure genetic homogeneity. While dilution plating yields spatially separated colonies, these may still contain microcolonies of different species, strain variants, or aggregated cells that jointly proliferate to form a visible single colony [14,15]. This phenomenon is particularly prevalent in environmental samples where syntrophic relationships or physical cell aggregation resist complete dispersal by dilution alone.

The streak plate method serves as the definitive purification technique to resolve this limitation. Unlike spread plating, which aims to separate cells across an agar surface for initial isolation, streaking progressively dilutes microbial biomass through continuous, directional dragging across fresh agar surfaces. By systematically flaming the inoculating loop between quadrants and reducing the inoculum density with each pass, cells are physically separated to the theoretical limit of single-cell deposition at the streak termini (Figure 1). Under suitable nutritional and environmental conditions, these isolated cells multiply to form discrete colonies with theoretically uniform genetic backgrounds, enabling researchers to pick these discrete colonies for pure culture establishment.

In practice, scientists frequently combine this agar-based purification with preceding techniques in a sequential workflow. In a typical procedure, environmental samples first undergo dilution to reduce cell density, followed by spread or pour plating to obtain initial separation. Alternatively, samples may be subjected to enrichment culture or selective media to amplify target populations. Regardless of the preceding method, subsequent streak purification remains essential to achieve monoclonality. For instance, Riham Surkatti et al. [32] examined process wastewater samples using LB media for initial enrichment, then applied the streaking method for definitive purification, successfully isolating three organic-degrading bacterial strains: *Alcaligenes faecalis*, *Stenotrophomonas* sp., and *Ochrobactrum* sp. Similarly, Marta N. Mota et al. [33] performed three rounds of enrichment on environmental samples, spread these enriched communities onto selective plates containing either glucose or xylose as the sole carbon source, and subsequently applied streak purification to acquire a pure strain of *Candida boidinii*.

Overall, agar-based streaking purification presents several distinct advantages. The operational workflow is mature and standardized, requiring minimal equipment and low experimental costs. The technique offers high versatility across microbial groups and provides direct morphological information, visible features like colony shape, size, color, and texture enable preliminary phenotypic identification prior to downstream genomic analysis. However, the method retains inherent limitations. The cultivation system exhibits significant selection bias, naturally favoring fast-growing, copiotrophic microbes while overlooking slow-growing colonies that may require extended incubation periods. Moreover, despite the theoretical basis of single-cell derivation, practical limitations exist: micro-colonies of different species may remain physically associated during streaking, or single colonies may arise from aggregated cell clusters, potentially introducing non-target contaminants and making it difficult to guarantee absolute monoclonality without repeated purification cycles. Consequently, for recalcitrant or slow-growing environmental isolates, streak purification may require multiple iterative rounds or combination with micromanipulation techniques (Section 3) to confirm clonality.

### 2.5. Special Technical Targets: Anaerobic Microorganisms and Extremophilic Microorganisms

Anaerobes and extremophiles show a much higher ecological dependence on the physicochemical parameters of their culture systems than conventional microbes. The core challenge in isolating and pure-culturing these microbes lies in one key factor. Researchers must strictly replicate and maintain the original ecological niche throughout the entire experiment.

When isolating strict anaerobes, simply incubating them without oxygen cannot guarantee survival. The fundamental strategy requires zero exposure to environmental oxygen throughout the whole process. Researchers must take strict measures to prevent oxygen interference at every single step [34]. These steps include environmental sampling, sample transport, single-cell sorting, and subculture recovery. Traditional aerobic experiments typically rely on shake flasks or agar plates. In contrast, acquiring anaerobes heavily depends on specific physical barriers to block oxygen. Laboratories commonly use equipment like anaerobic chambers, anaerobic jars, and sealed bottles filled with inert gases like nitrogen or carbon dioxide. Scientists must also add specific reducing agents to the culture medium to maintain a suitable growth environment. During actual operations, researchers frequently use the classic Hungate roll-tube method [35]. They also perform closed inoculations using strictly anaerobic hardware to sustain this oxygen-free environment.

This strict maintenance of in situ conditions equally applies to the cultivation of extremophiles. Culture systems for extreme microbes must continuously provide specific thresholds for temperature, salinity, pressure, pH, or redox potential [36]. When isolating thermophiles, the solidifying agents and nutrients in the medium must possess high thermal stability. This prevents them from degrading during high-temperature incubation. For instance, J. W. Deming et al. [37] developed a medium that remains solid at 120 °C and 265 atm. Using this, they successfully isolated hyperthermophilic bacteria from deep-sea hydrothermal vents in the Pacific Ocean. For extremely acidophilic or alkaliphilic strains, the culture system must offer a strong acid-base buffering capacity. Quehenberger et al. addressed this when culturing the thermoacidophilic archaeon *Sulfolobus acidocaldarius* [38]. They added citric acid to chelate iron ions and prevent precipitation. This addition also effectively improved the buffering capacity of the medium, solving growth inhibition caused by rising pH. Sampling and culturing deep-sea piezophiles present another major challenge. The decompression process, as samples move from the deep sea to the surface, can easily deactivate cells or trigger irreversible metabolic shock. To prevent this, researchers must rely entirely on pressure-retaining transfer devices and high-pressure bioreactors to complete all related operations.

Overall, while traditional methods (spread plating, selective media, enrichment and streaking purification for final clonal isolation) remain essential for physical isolation and enumeration, they collectively encounter a fundamental bottleneck when addressing microbial dark matter. They are biased towards fast-growing, culturable generalists and struggle to replicate the complex, oligotrophic conditions of natural habitats. To access the vast majority of uncultured microbial life, researchers are increasingly turning to novel, culture-independent, and single-cell technologies.

## 3. Advanced Methodologies for Microbial Isolation and Cultivation

As biotechnology develops, traditional plating methods are increasingly unable to meet the growing demand for accessing uncultured microorganisms. Consequently, a diverse array of novel isolation techniques has emerged to complement or supersede conventional approaches. This review categorizes these contemporary methods into several key domains: membrane diffusion-based culture, microfluidic technology, optical tweezers, fluorescence-activated cell sorting (FACS), Raman-activated cell sorting (RACS), and reverse genomics screening. The following sections provide a detailed examination of these technologies and their applications.

### 3.1. Mimicking Natural Environments via Membrane Diffusion

Membrane diffusion culture is designed to simulate the natural habitat of microorganisms, thereby successfully overcoming the limitations inherent in traditional static methods. It has emerged as a powerful tool for cultivating “uncultured” and “hard-to-culture” microbes [39].

The fundamental mechanism of the membrane diffusion method relies on a semi-permeable barrier. This barrier regulates the flux of substances between the enclosed microorganisms and the external environment [40]. By meticulously adjusting the pore size and material properties, researchers can selectively control molecular passage [41,42,43], creating optimal growth conditions for specific microbial groups. Many studies have demonstrated the versatility of this approach. For example, Hoess et al. designed self-supporting nanoporous aluminum oxide membranes [44], a setup that successfully induced specific gene expression in human stem cells during co-culture experiments. In the realm of environmental microbiology, Bollmann and colleagues successfully isolated bacterial strains from pond sediments that had previously proven recalcitrant to cultivation [45]. Similarly, Polrot utilized this technique to identify bacteria in sediments capable of resisting tributyltin (TBT) [46], identifying several specific species, including *Oceanisphaera* sp., *Pseudomonas* sp., *Bacillus* sp., and *Shewanella* sp. Furthermore, Modolon employed diffusion chambers and micro-well chip devices to culture microorganisms derived from coral mucus, leading to the isolation of several previously uncultured microbes, including nitrifying bacteria [47]. Membrane diffusion culture promotes archaeal growth by simulating their natural environments. Ding et al. used freshwater sediment as an inoculum [48] to successfully enrich a triple co-culture system within a hollow-fiber membrane biofilm reactor. Through this approach, a specific microbial community comprising DAMO archaea, DAMO bacteria, and anammox bacteria was established effectively.

**Figure 2:**
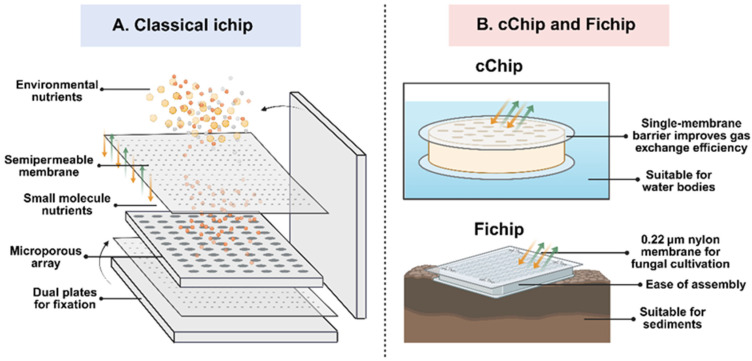
Schematic representation of high-throughput in situ cultivation devices. (**A**) The structure of the pioneering iChip, which utilizes a multi-well plate sandwiched between semi-permeable membranes. (**B**) Principles of simplified variants such as the cChip and FiChip, which optimize the assembly process and throughput for diverse environmental samples.

A significant advancement in this field was the development of the isolation chip (or “ichip”) by Brittany Berdy and colleagues [49]. They demonstrated that this device could effectively cultivate previously uncultured microbes from soil matrices. Adopting a similar strategy, Steinert et al. adapted the membrane diffusion method for marine environments [50], applying the technique to cultivate bacteria associated with sponges in their natural setting. Unlike traditional methods that expose microbes to high levels of nutrients, membrane diffusion allows nutrients to permeate slowly, maintaining a low-nutrient (oligotrophic) environment. This supports microorganisms that grow slowly or are sensitive to nutrient-rich substrates [51]. The membrane’s micropores are engineered to allow the free passage of small molecules (such as salts, amino acids, and dissolved oxygen) while effectively blocking macromolecules like proteins and polysaccharides, as well as competing bacteria. This selectivity ensures that target microbes have a dedicated space for proliferation. Furthermore, the semi-permeable nature allows metabolic waste products to diffuse out, preventing the accumulation of toxic byproducts (Figure 2). This equilibrium mimics natural conditions, which is the key factor driving the method’s success. Research by Kaeberlein indicated that this technique could recover up to 40% of bacteria from marine sediments [39], a stark contrast to the 0.05% recovery rate of standard plates. This evidence confirms that membrane diffusion significantly enhances the culturability of recalcitrant microbes. Further innovations include Meng’s development of a fungal enrichment method and a “FiChip” for mangrove sediments [52], and Lodhi’s “cChip” which improves efficiency by facilitating more direct environmental contact than the original iChip [53], leading to the discovery of 45 new microbial species. Additionally, Zhao modified the chip design to successfully domesticate microbes from extreme hot spring environments [54].

For microorganisms living in extreme environments, membrane diffusion culture offers conditions that closely resemble their natural habitat. During the isolation of anaerobic microorganisms, this method reduces oxygen exposure by better preserving natural environmental conditions. Microorganisms can also use semipermeable membranes to obtain growth factors, nutrients, and metabolic signals from the surrounding environment. The membrane diffusion culture method helps alleviate oxygen stress and, to some extent, reduces the competitive advantage of fast-growing contaminants found in traditional cultures. Scientists face several practical problems despite these benefits. Device assembly becomes complex under anaerobic conditions, and gaseous substrates often lack sufficient diffusion. Researchers find it difficult to observe or purify anaerobic colonies. Directed pure cultivation of syntrophic consortia remains another major challenge. Consequently, this approach primarily suits small scale research. Such research focuses on the discovery of high value anaerobic taxa and the exploration of microbial diversity.

The primary advantage of the diffusion chamber method is its ability to substantially improve the cultivation of “unculturable” microbes. By leveraging the semi-permeable membrane, the system facilitates the influx of essential environmental nutrients while simultaneously allowing the efflux of metabolic waste. This exchange prevents toxicity and maintains an oligotrophic environment, enabling researchers to grow difficult strains in situ. However, the technology is not without limitations. The cultivation cycle is often protracted, requiring weeks or even months. Furthermore, the method is generally low-throughput and cannot process large sample volumes simultaneously. Efficiency is also heavily dependent on the membrane material, and the development of novel, biocompatible materials can be cost-prohibitive. In practice, this technology relies on expensive semipermeable materials and precise chip devices. The experimental cycle takes a long time, and most setups are designed for a single use. These factors drive the isolation cost per sample much higher than traditional methods. Membrane materials also demonstrate significant differences in stability and adaptability across various environments. This inconsistency prevents the establishment of a standardized platform. Membrane diffusion culture generally favors oligotrophic and slow growing microorganisms. However, it remains limited in its capacity to support the growth of strictly syntrophic or rapidly proliferating strains. Therefore, specific parameters, including membrane material and pore size, must be carefully selected. This selection often introduces a selective enrichment bias. Multiple cell types might also be mixed within the initial inoculum. Scientists therefore still need to perform subsequent purification steps.

In conclusion, the membrane diffusion method utilizes a semi-permeable interface to regulate substance exchange, effectively circumventing the “Great Plate Count Anomaly”. It is particularly well-suited for isolating specific functional strains and simulating natural growth conditions, finding widespread application in diverse environments such as soil, sediments, oceans, and geothermal springs. Nevertheless, compared to high-throughput separation methods, it operates at a slower pace, making it less effective for large-scale screening or the rapid acquisition of vast numbers of pure strains. Despite these constraints, membrane diffusion remains a milestone technology, successfully breaking the “pure culture bottleneck” and enabling the direct domestication of uncultured strains from the environment.

### 3.2. Microfluidics-Based Cell Separation and Sorting

Microfluidic technology provides a new solution for isolating bacterial strains, capitalizing on advantages such as miniaturization, automation, and high throughput. By precisely controlling fluid behavior within micro-scale channels, this method achieves the efficient and rapid separation of microorganisms [55]. The process exploits differences in the physical properties of microbes (such as size, shape, deformability, and surface charge) as well as biological distinctions like surface antigens and genetic composition. Separation is accomplished either through the specific structure of the micro-channels or by applying external forces. Based on the different mechanisms of action, this review categorizes microfluidic methods into two primary classes: active microfluidics and passive microfluidics [56,57,58].

#### 3.2.1. Passive Microfluidics

Passive microfluidics operates without the requirement for external energy fields. This category is further divided based on fluid mechanics into inertial microfluidics, which relies on channel geometry and fluid inertia, and viscoelastic microfluidics, which utilizes the viscoelastic properties of the fluid. These methods leverage channel design and hydrodynamic forces to sort cells based on physical traits, enabling label-free separation. Currently, this approach represents the dominant strategy for passive microbial separation.

**Figure 3:**
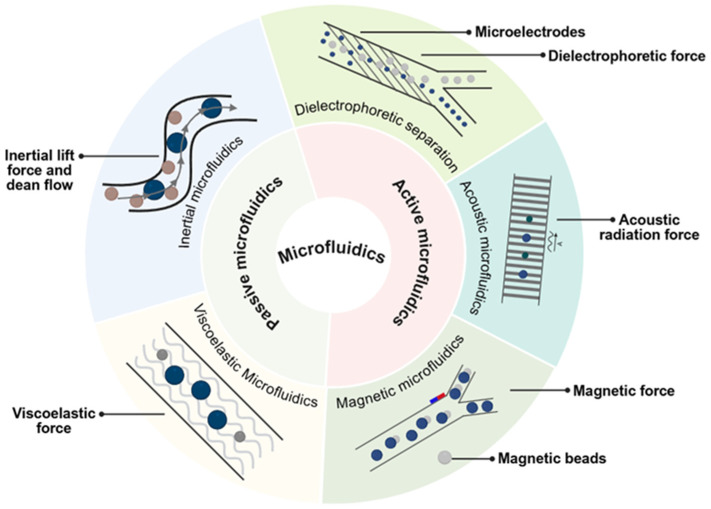
Classification of microfluidic cell separation technologies. The diagram distinguishes between passive microfluidics, which rely on intrinsic hydrodynamic forces (e.g., inertial lift force, viscoelastic force) and channel geometry, and active microfluidics which utilize external energy fields (e.g., dielectrophoretic, acoustic, and magnetic forces) for precise cell manipulation and sorting.

Inertial microfluidics utilizes inertial lift forces and Dean flow induced by channel curvature (Figure 3). These hydrodynamic forces compel microbes of varying sizes to migrate along distinct trajectories, thereby achieving separation. This technique achieves high-throughput, size-based separation using only the microchannel structure, with resolution largely dependent on size differentials [59]. For example, Condina employed spiral microchannels to rapidly separate spoilage microbes from background yeast in beer, achieving over 90% efficiency and a throughput of 2.7 × 10^6^ cells/min [60]. Similarly, Barbosa designed a chip utilizing deterministic lateral displacement (DLD) to separate yeast based on size and tilt angle [61].

Viscoelastic microfluidic technology achieves separation by adjusting the flow properties of the carrier fluid within the micro-channels [44] (Figure 3). This approach offers significant advantages in manipulating particles across a broad size range, from microns down to nanometers. It supports high-throughput operations, with flow rates ranging from microliters per hour to milliliters per minute [62]. Liu Ping utilized both viscoelastic and standard fluids to separate yeast based on imorphology [63], while Zhang Tianlong successfully separated bacteria according to size using this technique [64]. In clinical applications, Lim Hyunjung applied the technology to isolate and purify Candida cells from blood samples [65]. Furthermore, Liu Ping demonstrated that the method could enrich and separate *Bacillus subtilis* strains based on cell length [66].

#### 3.2.2. Active Microfluidics

While passive microfluidic methods rely solely on channel geometry or fluid properties, active microfluidic technology integrates external energy fields as its core mechanism. By applying precisely controllable forces to specific microorganisms, this method overcomes the limitations of passive systems. It offers better specificity and operational flexibility, allowing for the selective isolation of strains. Active techniques primarily employ acoustic, electric, or magnetic fields to separate microbes based on physical or biological differences, such as dielectric properties or density. This article categorizes active microfluidic technology into dielectrophoretic, acoustic, and magnetic separation techniques.

Dielectrophoresis (DEP) separation technology functions by utilizing non-uniform alternating current (AC) electric fields to manipulate particles. In these fields, different particles experience distinct forces, facilitating separation. Microorganisms possess unique dielectric properties due to variations in membrane composition, internal structure, and size, resulting in differential DEP forces under identical electric field conditions [67,68,69]. To generate these non-uniform fields, DEP devices typically incorporate arrays of micro-electrodes (Figure 3). Depending on the frequency applied and the specific traits of the microbe, cells may exhibit positive DEP (moving toward high-field regions) or negative DEP (moving away). Precise separation is achieved by meticulously controlling field parameters and micro-channel design [67]. Moncada-Hernandez et al. utilized insulator-based DEP to concentrate and separate bacteria from yeast [69], while Yu employed DEP to purify ocean bacteria [70]. In clinical contexts, Thomas successfully separated E. coli from blood [71], and Ganesan isolated *Staphylococcus aureus* [72]. Currently, this technology is capable of handling high cell volumes; for instance, Gao and colleagues achieved high-speed separation of live and dead yeast cells with a throughput of 1.79 × 10^5^ cells per minute [73]. Chen et al. activated a DEP microfluidic chip using nanoelectrodes [74]. This device achieved highly selective capture and quantification of spores from Sclerotinia sclerotiorum, an airborne pathogenic fungus. The cell membrane composition of archaea differs from that of bacteria and fungi, and this structural difference leads to a unique dielectric response. This distinct property is widely utilized as a key basis for isolating archaea via DEP. Guided by this principle, Mogi et al. applied DEP for single-cell manipulation. Through their system, *Haloferax volcanii*, an extremely halophilic archaeon, was successfully captured and separated [75].

Acoustofluidics integrates acoustic principles with microfluidic to enable label-free, non-contact manipulation of micro- and nano-scale particles [76]. This technology leverages acoustic radiation forces to achieve selective separation based on differences in size, density, compressibility, and acoustic contrast (Figure 3). The approach offers distinct advantages, including excellent biocompatibility and gentle operation [77]. Ai et al. established a standing surface acoustic wave (SSAW) field to separate *E. coli* from blood [78]. Using a modified approach, Lee applied tilted-angle standing surface acoustic waves (taSSAW) to successfully separate *E. coli* from platelets [79]. Ding Xiaoyun developed a similar technology achieving a throughput of 1 × 10^4^ cells/min [80], while Ang Bryan fabricated a micro-scale particle bed to capture bacterial cells via acoustic excitation, enabling rapid detection in milk [81]. Byeong Seon Park et al. coupled acoustofluidics with pinched flow fractionation. This integration built a label-free and highly efficient microfluidic separation platform, requiring no sample pretreatment. Within this device, the distinct sizes and acoustic responses of fungal spores and eukaryotic cells are effectively utilized. This mechanism enables the continuous and high-purity isolation of typical fungal spores, such as *Penicillium* and *Aspergillus*, from eukaryotic cells. Through this approach, a separation efficiency of over 90% is achieved [82].

Magnetic microfluidic microbial separation integrates immunomagnetic separation with chip platforms. It employs magnetic nanoparticles (MNPs) surface-modified with recognition molecules, such as antibodies or aptamers. These particles selectively capture target microorganisms, forming microbe-bead complexes. Subsequently, an external magnetic field exerts force on these complexes, enabling their spatial separation and enrichment within microchannels [83,84] (Figure 3). While more common in mammalian cell separation, applications for microbial isolation are emerging. Inglis et al. designed a device to separate magnetically labeled cells from blood [85]. Jo Younggeun utilized magnetic particles within droplets to sort cells based on magnetic force [86], and Thi developed a chip to remove tumor cells from whole blood [87].

For anaerobic bacteria, microfluidic platforms utilize microscale closed systems, droplets, or microchambers. These designs control oxygen diffusion, nutrient gradients, and metabolite exchange very precisely within small volumes. Such precise control helps to reduce the risk of oxygen exposure and creates a near-natural microenvironment for slow-growing, low-abundance, or highly ecologically dependent microbes. For extremophiles, microfluidic technology allows researchers to regulate parameters like temperature, salinity, pH, pressure, or substrate concentration with high accuracy. The method supports high-throughput screening and single-cell-resolution analysis. This capability makes it especially suitable for discovering rare, hard to culture, or uniquely adapted microbial groups from complex environmental samples. However, materials such as PDMS exhibit nonspecific adsorption and gas permeability. These properties can alter the chemical composition of the microenvironment. Researchers must therefore equip the system with strictly anaerobic hardware to study and isolate strict anaerobes.

Passive techniques offer the advantage of simplicity, requiring no complex external energy and relying on robust physical differences. However, they struggle with target strains that are physically similar but taxonomically distinct. Active microfluidics introduces external energy fields for precise, controllable manipulation, excelling in handling complex samples. Yet, high performance often entails increased system complexity. Since a single mechanism rarely satisfies all requirements for throughput, resolution, and specificity, the current trend is moving toward hybrid microfluidics, which combines active control with passive structures for enhanced integration and precision. Overall, the development of microfluidic equipment and chips requires a high cost. Both system operation and data analysis demand specialized technical support. The technology offers high throughput. However, no unified standards have been established for diverse chip platforms. This inconsistency limits reproducibility between laboratories and restricts scaled up applications. Microscale closed environments tend to favor microorganisms capable of growing in small volumes, under low shear stress, or within specific droplets. Sample pretreatment might also damage fragile cells. It can easily disrupt natural symbiotic relationships among microbes.

In summary, microfluidic technology couples micro-scale fluid mechanics with physical fields to achieve customized, automated sorting while maintaining biological viability. It effectively addresses the low throughput and resolution issues of traditional methods. However, separation efficiency often relies on physical or labeled differences, limiting the distinction of phenotypically similar but functionally distinct microbes. Therefore, microfluidics currently serves best as a high-throughput “front-end screening tool” rather than a final isolation method.

### 3.3. Optical Trapping and Manipulation Techniques

In 1986, Ashkin invented the single-beam gradient force optical trap, marking the inaugural application of optical tweezers in biology [88]. Since then, optical tweezers have evolved into a critical tool for single-cell microbial separation. This technology is prized for its ability to manipulate samples with extreme precision without physical contact, ensuring minimal mechanical damage [89]. It operates by using highly focused laser beams to generate optical traps capable of capturing and moving particles ranging from micrometers to nanometers [88]. Mitchell et al. utilized this method as early as 1993 to isolate single bacteria from complex communities [90]. Today, optical tweezers are frequently combined with detection tools like Raman spectroscopy and fluorescence, or integrated with microfluidics (e.g., the OPSI system by Xu Teng [91]) to screen and isolate single cells such as *Nitrospira* bacteria [92]. This review classifies optical tweezers into three primary categories: holographic techniques, vortex beams, and laser-induced forward transfer (Table 1).

#### 3.3.1. Holographic Optical Tweezers

Holographic Optical Tweezers (HOT) function by employing a Spatial Light Modulator (SLM) to modulate the wavefront of the incident light. This process generates a specific light field pattern in the focal plane, enabling the assembly and manipulation of multiple microscopic samples [93]. By loading computer-generated holograms onto the modulator, a single beam is split into multiple independent beams, forming an array of optical traps. Users can dynamically adjust the position and number of these traps in real-time [94]. Unlike traditional tweezers, HOT supports the parallel capture of multiple targets. Lafong et al. constructed a system to control multiple traps independently, handling yeast and fungal spores [95]. Hoerner utilized the technology to arrange *Bacillus subtilis* [96], while Werner combined HOT with microfluidic chips to hold over two hundred yeast cells simultaneously without contact [97].

#### 3.3.2. Vortex Beam Optical Tweezers

A vortex beam is a structured light field characterized by a helical wavefront and a phase singularity. It enables the stable trapping of particles with refractive indices either higher or lower than the surrounding medium [98]. A defining feature is the carriage of orbital angular momentum; when the light interacts with a particle, it transfers this momentum, applying a torque that causes the trapped object to rotate around the beam axis [99]. In microbial separation, this technology exploits the differential response of cells to the light field. By adjusting laser power and fluid velocity, researchers can selectively manipulate specific cells [100]. Zhang Peng applied vortex beam optical tweezers to achieve the stable trapping and controlled rotation of *E. coli* [101].

#### 3.3.3. Laser-Induced Forward Transfer

Laser-induced forward transfer (LIFT) utilizes a pulsed laser to propel donor material from a source substrate to a receiving substrate. In microbial isolation, the donor material is irradiated, and the laser energy pushes a portion of the material (containing microbial cells) onto the receiver, achieving both isolation and transfer [102]. LIFT offers high-resolution and non-contact deposition, functioning effectively across various environmental conditions [103]. Crucially, it allows for the precise transfer of single cells. Cheptsov used hydrogel droplets to separate yeast [104], while Liang developed a version for accurate single-cell separation [105]. Wang integrated LIFT with a microwell chip, achieving high-speed processing of tens of thousands of cells while maintaining viability [106]. Chen further improved the technology using optical vortex LIFT (OV-LIFT) and flat-top LIFT (FT-LIFT), successfully recovering *Saccharomyces cerevisiae* and *Escherichia coli* with enhanced precision [107].

**Table 1:** Comparison of different optical tweezers techniques.

Technology	Throughput	Core Mechanism	Key Advantages
Holographic optical tweezers	**Medium** (tens to hundreds of cells [97])	Uses Spatial Light Modulators (SLM) to generate multiple independent traps.	**3D Parallelism:** Arbitrary trap arrangement in 3D space for simultaneous multi-cell manipulation.
Vortex beam optical tweezers	**Low** (single or a few cells [101])	Carries orbital angular momentum to apply torque to particles.	**Rotational Control:** Enables stable rotation of cells; ideal for studying cellular mechanics and viscosity.
Laser-induced forward transfer	**High** (thousands of cells [106])	Uses laser pulses to induce cavitation bubbles, ejecting cells from a donor film.	**High-speed Transfer:** Contactless isolation of specific cells; versatile for solid/liquid substrates.

For anaerobic bacteria, optical tweezers help reduce the damage caused by repeated transfers and air exposure during traditional plate isolation. When studying extremophiles, researchers can directly manipulate single cells under conditions that closely resemble the original liquid environment. This capability makes the technique ideal for isolating rare or hard to culture individuals from complex backgrounds. Such environments often feature high salinity, low nutrients, or extreme pH levels. As a high-resolution and target-oriented capture tool, optical tweezers precisely isolate specific single cells. Scientists use this method to connect the initial isolation of anaerobes or extremophiles with their subsequent cultivation.

The paramount advantage of optical tweezers is the ability to manipulate particles from micrometers to nanometers without physical contact, avoiding the mechanical stress associated with traditional methods. Holographic tweezers facilitate multi-target capture; vortex beams introduce rotational manipulation via orbital angular momentum; and LIFT offers high-resolution printing (Table 1). However, limitations exist. High-intensity lasers can induce photodamage if not carefully regulated. Optical sorting relies heavily on microscopic observation. Researchers identify targets based on cell morphology, size, and motility. They may also use additional detection signals to pinpoint their desired cells. Microbes with unclear shapes, however, often blend in with background particles. Other cells show high sensitivity to laser irradiation and easily suffer damage during manipulation. As a result, these specific microorganisms might escape detection or remain underestimated in scientific studies. The manual selection process itself introduces an additional subjective bias based on operator judgment. The technology necessitates precise optical systems, resulting in high costs and technical barriers. These limitations prevent optical tweezers from achieving the widespread popularity of conventional cultivation methods. The technology undoubtedly offers exceptional single-cell resolution. Its core advantage, however, remains precise manipulation rather than large scale parallel processing. Furthermore, separating specific cells from complex, dense communities remains challenging. Consequently, optical tweezers are most effective when combined with other technologies, such as Raman spectroscopy or FACS, serving primarily as a precise capture tool rather than a standalone analytical method.

### 3.4. Fluorescence-Based High-Throughput Sorting

**Figure 4:**
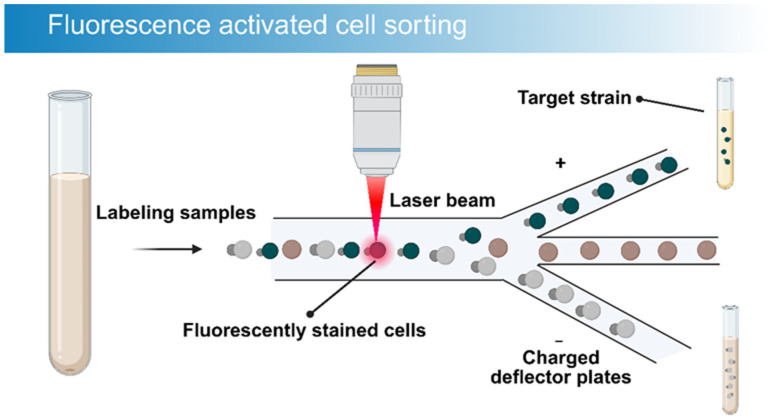
Schematic diagram of the Fluorescence-Activated Cell Sorting (FACS) process.

First proposed in 1969 [108], fluorescence-activated cell sorting (FACS) has evolved into a robust instrument for single-cell analysis and separation. Combining flow cytometry detection with cell sorting capabilities, FACS allows for multi-parameter analysis and the precise isolation of single cells under high-speed flow conditions, processing up to 10^7^ to 10^8^ cells per hour [109]. This exceptional speed accelerates the discovery of rare microbes. FACS sorts cells individually, ensuring strain purity and eliminating the heterogeneity of mixed cultures. Moreover, the system supports multi-parameter detection, simultaneously assessing multiple cellular characteristics to enhance specificity. Beyond sorting based on intrinsic features, FACS utilizes fluorescent probes to target microbes with specific functional attributes [110]. The fundamental mechanism involves hydrodynamic focusing to align cells in a single file. As cells traverse a laser beam, scattered light and fluorescence signals are detected, and target cells are sorted based on preset parameters [111] (Figure 4).

Applications of FACS in microbial research are expanding. Skrekas linked metabolite levels to fluorescence, enabling the high-throughput screening of libraries for high-yield genotypes [112]. Ahn et al. utilized FACS to screen randomly mutated bacteria for phosphate storage, isolating *Lactobacillus paraplantarum* SNUP7 [113]. Gemma et al. employed FACS-uORF to measure thousands of yeast uORFs simultaneously [114,115]. Wang’s group combined CRISPR/Cas9 with reporter genes (mCherry, GFP) to isolate specific recombinant viruses [116]. Additionally, Tan employed an ultra-high-throughput FACS method based on lactose permease (LacY) specificity to distinguish fluorescent glycosylation products [117]. Sallet et al. developed a high-throughput culture system for anaerobic microbes [118]. Their platform combines hydrogel microcapsules with fluorescence-activated cell sorting (FACS). Hard-to-culture soil anaerobes, such as sulfate-reducing bacteria, were efficiently isolated using this technique. This modern approach significantly improves the overall culturability of anaerobic microorganisms. Hatzenpichler and colleagues combined BONCAT with FISH and FACS. They used this approach to sort and identify anaerobic methanotrophic archaea and sulfate-reducing bacteria from deep sea methane seep sediments. Their study also reported, for the first time, a novel symbiotic consortium formed by ANME and *Verrucomicrobia* [119].

In anaerobic microbiology, Fluorescence-Activated Cell Sorting (FACS) improves the recovery efficiency of target taxa. Researchers achieve this through a strategy of encapsulation and cultivation prior to sorting. For extremophiles, FACS rapidly identifies and isolates specific cells within large populations. Scientists use fluorescence signals to detect particular functional states. The technique effectively screens low abundance targets from complex environmental samples, especially when paired with metabolic probes or prior enrichment. The success of this approach depends on a few key factors. Cells must maintain their viability before and after sorting. They also need to accept stable labels or generate detectable signals. Strict anaerobes require specialized culture and transport systems. These setups must minimize oxygen exposure as much as possible.

The primary benefit of FACS is its capacity to process massive cell volumes while maintaining single-cell precision. It performs multi-parameter detection, checking static markers as well as dynamic functional states like enzyme activity and metabolism. However, the technology relies heavily on fluorescent markers. The introduction of exogenous dyes can potentially alter cellular activity or induce cytotoxicity. Researchers must know the physiological traits of target microbes in advance to design effective probes. This requirement prevents the unbiased discovery of completely new populations. Factors like symbiotic dependencies and specific nutritional needs often restrict the success rate of pure cultivation. Consequently, the recovery of strains after sorting remains a significant challenge.

In summary, FACS serves as a cornerstone screening tool. It efficiently links phenotypes to genotypes via fluorescent signals, significantly accelerating the discovery of functional genes and superior strains. Its unique combination of ultra-high speed, precision, and single-cell resolution makes it indispensable. Future developments must focus on enhancing cell viability and developing non-toxic probes. Ultimately, FACS addresses the throughput limitations of traditional methods, acting as a powerful accelerator for high-throughput screening.

### 3.5. Raman Spectroscopy-Based Single-Cell Sorting

**Figure 5:**
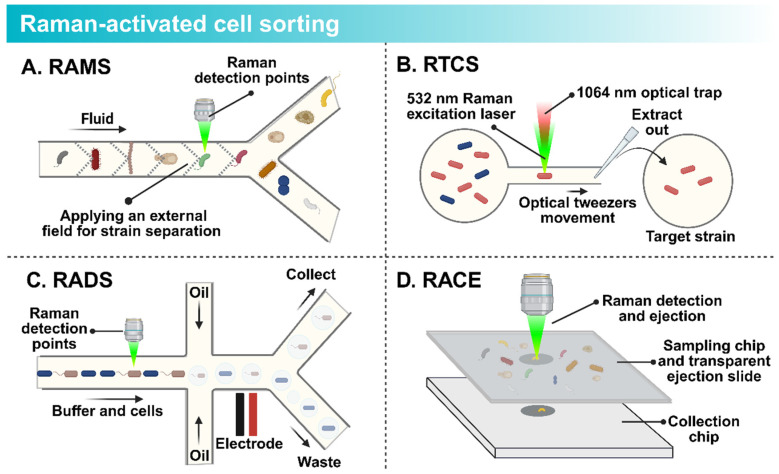
Schematic illustration of representative Raman-activated cell sorting platforms. (**A**) Raman-activated Microfluidic Sorting (RAMS); (**B**) Raman Tweezers Cell Sorting (RTCS); (**C**) Raman-activated Droplet Sorting (RADS); (**D**) Raman-activated Cell Ejection (RACE).

The development of sensitive non-resonant Raman spectroscopy by Puppels et al. in 1990 [120] paved the way for Raman-activated Cell Sorting (RACS). This innovation method sorts single cells without the need for extrinsic labels. As cells flow past a laser detection point, the system analyzes the Raman spectrum of each cell in real-time. Upon identifying a target, the cell is directed into a collection channel (Figure 5). RACS utilizes the Raman spectrum as a “chemical fingerprint” revealing the cell’s internal composition (proteins, DNA, lipids, and carbohydrates) [121]. This intrinsic data provides deep insights into the cell’s physiology and metabolic state. Recent advancements in spectral acquisition speed, coupled with microfluidics, have led to breakthroughs in throughput [122]. Tang et al. classified these technologies into four main types [123]: Raman-activated microfluidic sorting (RAMS), Raman tweezers cell sorting (RTCS), Raman-activated droplet sorting (RADS), and Raman-activated cell ejection (RACE).

RAMS integrates Raman detection directly with microfluidic chips for high-speed sorting (Figure 5A). Lindley et al. combined a broadband FT-CARS spectrometer with a push-pull sorter, achieving a theoretical speed of 3000 cells/min for sorting microalgae [124]. Wang et al. developed a system using positive dielectrophoresis (pDEP) to capture and sort fast-moving single cells based on functional properties [125,126].

RTCS employs optical tweezers to trap cells for analysis. One laser holds the cell while another acquires the spectrum (Figure 5B). Lee et al. constructed an automated platform using this technology [127], and Fang et al. successfully separated target microbes in a reservoir [128]. Later, Lee developed a fully automated system for metabolic sorting, reaching a throughput of 3.3 to 8.3 cells/min [129].

RADS combines droplet microfluidics with Raman detection, encapsulating single cells in oil droplets (Figure 5C). Wang designed a RADS system to screen *Haematococcus pluvialis* for astaxanthin production, achieving a sorting rate of ~260 cells/min [130]. Jing developed a gravity-driven system that uses D_2_O labeling to track metabolic activity, enabling the sorting and sequencing of low-abundance soil bacteria with true single-cell resolution [131].

RACE is based on laser-induced forward transfer (Figure 5D). Cells on a donor slide (coated with an energy-absorbing layer) are identified by their spectral signature. A pulsed laser then vaporizes the layer beneath the target, ejecting the cell into a collector. Jing designed an integrated RACE device to isolate CO_2_-fixing marine bacteria [132]. While traditional RACE is slow, Zhang developed S-RACE, integrating stimulated Raman imaging to achieve a throughput of ~756 cells/min for isolating lipid-rich *Rhodotorula glutinis* [133]. Wang and colleagues integrated single-cell Raman spectroscopy with artificial intelligence-based support vector machine classification and Raman-activated cell ejection (RACE). This strategy enabled highly accurate identification of nine cultured archaeal strains at the single-cell level. In the same study, the authors used Raman-fluorescence in situ hybridization (Raman-FISH) to obtain in situ Raman spectra of uncultured marine MGII archaea. These spectra revealed their distinct lipid biosynthesis feature [134].

For anaerobic bacteria, researchers can combine RACS with microfluidic cultivation, closed droplets, or anaerobic workstations. This integration allows the system to identify target cells with specific metabolic activities before directing the sorting process. Such a strategy reduces the cellular damage caused by repeated oxygen exposure during traditional isolation. The method excels at screening slow-growing and highly ecologically dependent microbes. It also benefits the study of extremely rare taxa with specific functions. When studying extremophiles, RACS evaluates single cells under conditions that closely mimic their native habitats. Scientists use this tool to analyze cells derived from environments with high salinity, extreme pH, low nutrients, or other complex matrices. RACS is better suited as a function-oriented tool for the discovery and targeted isolation of anaerobic bacteria and extremophiles.

Raman spectroscopy offers distinct advantages: it is label-free, non-destructive, and capable of in situ analysis. It avoids artificial interference from dyes, preserving sample integrity for downstream cultivation. Furthermore, it provides multidimensional data on biological molecules simultaneously. Researchers are increasingly combining RACS with artificial intelligence to improve accuracy [135]. While current studies link genotypes to phenotypes [136], future integration with single-cell sequencing could fully bridge the phenotype-genotype gap. However, challenges remain, including weak natural Raman signals in some strains and high technical complexity. The system primarily recognizes cells with clear Raman signals. It also readily detects microbes that produce sufficient spectral differences under specific substrate or isotope-labeling conditions. This inherent reliance naturally favors metabolically active populations. These favored groups usually display strong signals or contain abundant marker molecules. Conversely, target cells with low metabolic rates or weak spectra often escape detection. Small cell sizes and complex background matrices can also obscure these specific microbes. Practical factors, including laser irradiation and sample preparation methods, further impact cell viability. Environmental controls similarly alter the stability of the resulting Raman spectra.

Despite these challenges, label-free sorting based on single-cell Raman spectroscopy has become a critical tool. It bridges the gap between microbial traits and functions, allowing for the precise separation of target cells from mixed communities. Compared to traditional methods, RACS offers an irreplaceable advantage in analyzing cellular metabolic phenotypes and serves as a vital link to downstream genomic analysis.

### 3.6. Genome-Directed Reverse Isolation Strategies

With the proliferation of high-throughput sequencing, metagenomics and single-cell genomics have generated vast datasets, significantly expanding our comprehension of microbial diversity. A central challenge in modern microbiology is utilizing this genetic information to guide the cultivation of the “microbial dark matter” (the vast majority of microbes that remain uncultured yet play critical roles in ecosystems). Sequencing data can be obtained through metagenomics or single-cell genomics. After further assembly processing, such data enables the reconstruction of genomes from target microorganisms. These highly complete draft genomes reveal the evolutionary position of the microbes, along with their metabolic potential and survival strategies. Functional annotation gives biological meaning to these sequences. Scientists compare the data against public databases, such as KEGG. This step helps identify genes related to key metabolic pathways and nutritional needs, as well as environmental adaptation and signal transduction [137]. Such genomic analysis accurately predicts microbial nutritional needs by annotating metabolic genes. Researchers can find out, for instance, whether a microbe lacks the ability to produce specific amino acids, vitamins, or cofactors. They can then directly add these missing nutrients to the culture medium. The environmental tolerance and preferences of these microbes are also revealed through genomic analysis. This information provides a reliable basis for designing suitable culture conditions [138]. Another study used single-cell genomics to guide cultivation. With this strategy, researchers successfully isolated the first terrestrial nanoarchaeon, *Nanopusillus acidilobi*, together with its host, *Acidilobus* sp. 7A, from a terrestrial geothermal environment [139]. Researchers can identify potential cross-feeding relationships to build co-culture systems, which simulate the ecological niches required for natural microbial growth.This genome-based design strategy significantly improves the success rate of isolating hard-to-culture microbes. The concept of reverse genomics perfectly represents this research approach [140].

“Reverse genomics” proposes a novel strategy to address this bottleneck. Proposed by Karissa et al. in 2019 [141], this approach inverts the traditional workflow, moving from genome to culture. Researchers utilize the genomic data of a target microbe to predict specific surface markers or antigens. Subsequently, custom probes (such as antibodies or nucleic acid tags) are synthesized to target these markers, enabling the isolation of specific microbes from complex communities. Using this technology, the team successfully isolated three types of TM7 bacteria from the human oral cavity, despite their extreme rarity. This technology is equally effective for environmental samples. Li et al. developed a method combining RACS, Stable Isotope Probing (SIP), and Genome-Directed Cultivation (GDC) [142] to identify and culture active toluene-degrading bacteria in polluted soil. By analyzing genes related to metabolism and transport, they designed a specific culture medium, successfully domesticating the target strains.

Reverse genomics uses genomic annotations to identify specific microbial needs. Based on this information, researchers can supply the special coenzymes, anaerobic factors, and reducing conditions essential for anaerobes. For extremophiles, the method accurately matches harsh in situ parameters, such as temperature, salinity, pH, and pressure. Specific probes can also be designed in advance. These probes bind directly to the target microorganisms. As a result, the isolation success rate for hard-to-culture anaerobes and extremophiles is significantly improved. Reverse genomics significantly enhances the success rate of culturing unknown microbes. By sequencing sorted bacteria, scientists can decipher specific metabolic requirements, allowing for the rational design of culture media rather than relying on trial-and-error. The technique offers high specificity, precisely identifying microbes with distinct functions. However, it relies heavily on the availability of accurate genomic data and faces technical challenges in probe development. The preparation of specific probes incurs significant costs. This financial barrier makes the technique impractical for large-scale general screening. The overall workflow also suffers from a low level of standardization. To achieve success, researchers must rely heavily on the existing genomic data of target species and the efficiency of probe development. Such strict requirements naturally limit the scalability of the method. The system therefore struggles to quickly adapt to completely unknown microbial taxa.

In conclusion, reverse genomics shifts the paradigm from random attempts to rational design. Genomic data serves not merely as an analytical output but as a blueprint for isolation. This approach is ideal for targeting microbes with known genomes but unknown culture conditions. Fundamentally, reverse genomics transforms the logic of microbial isolation, transitioning the field from “attempted culture” to “designed culture” and serving as a vital bridge between genomic big data and living microbial resources.

## 4. Conclusion and Outlook

This review summarizes the evolution of microbial isolation and culture techniques, highlighting a distinct paradigm shift from traditional population-based growth to modern single-cell manipulation and analysis. Traditional approaches, such as spread plates, selective media, and enrichment cultures, remain foundational but are fundamentally limited by their reliance on artificial conditions, recovering only a minute fraction of microbial diversity.

As the field has advanced, innovative techniques have emerged to address these limitations. Membrane diffusion culture represents a significant leap forward, successfully mimicking natural environments by controlling nutrient diffusion. Conversely, single-cell technologies (including microfluidics, optical tweezers, FACS, and RACS) have revolutionized the field by decoupling “isolation” from “cultivation”. These methods identify and separate specific cells from complex communities based on physical traits, chemical composition, or specific markers, independent of their immediate ability to grow on agar. Furthermore, reverse genomics has introduced a targeted approach, utilizing existing genetic data to design specific isolation strategies. A detailed comparison of these techniques is presented in Table 2 and Table 3.

Fully uncovering microbial “dark matter” remains a formidable challenge. As each isolation technique possesses distinct strengths and limitations, the future of the field lies in the integration of complementary technologies into unified platforms. Based on the principles discussed, we propose the following methodological strategies: (i) *For uncultured microbes in complex samples* (e.g., *soil*): We recommend combining in situ membrane diffusion culture with high-throughput sorting. For instance, Liu et al. successfully combined FACS with the iChip to access uncultured soil microbes [143]. (ii) *For discovering new natural products:* A combination of reverse genomics screening and microfluidic sorting is advisable to target specific gene clusters. (iii) *For isolating low-abundance active cells with known functions:* Researchers should combine Raman-activated cell sorting (RACS) with microfluidic cultivation to identify cells based on metabolic activity (e.g., substrate degradation) before isolation.

Despite these advancements, significant technical and biological hurdles remain. Currently, a major trade-off exists between throughput and resolution. Technologies that offer high-resolution phenotypic analysis, such as Raman-activated cell sorting (RACS) and optical tweezers, often suffer from low throughput and high operational complexity, making them unsuitable for large-scale bioprospecting. Conversely, high-throughput methods like FACS often require labeling, which can alter cell physiology or fail to detect novel taxa lacking known markers. Therefore, the immediate technological imperative is the development of automated, high-throughput, label-free platforms. The integration of Artificial Intelligence (AI) and Machine Learning (ML) into these systems represents a critical frontier. AI-driven image recognition and real-time data processing can drastically increase sorting speeds and accuracy, enabling the intelligent selection of target cells based on complex morphological or metabolic signatures without human intervention.

Furthermore, physical isolation is merely the preliminary step; the biological challenge of “post-isolation survival” is often the true bottleneck. Many environmental microbes are auxotrophic or rely on syntrophic interactions, meaning they cannot survive in isolation due to the absence of essential signaling molecules or metabolic cross-feeding provided by their community. Consequently, “successful isolation” does not guarantee “successful culture”. Future research must pivot from simple physical separation to niche-mimicking cultivation. This involves developing micro-bioreactors that not only isolate single cells but also precisely control micro-environmental factors (such as oxygen gradients, signaling peptides, and co-culture partners) to trigger resuscitation and sustain growth. Strategies that bridge the gap between single-cell genomics and culture, such as using genomic data to predict nutritional requirements (reverse genomics), will be essential for reviving these “viable but non-culturable” (VBNC) states.

In the future, advances in microbial isolation and cultivation will rely on the deep integration of multi-omics approaches, culturomics, and artificial intelligence (AI). Multiomics technologies, by integrating information from metagenomics, transcriptomics, proteomics and metabolomics, can more comprehensively reveal the composition, metabolic potential and ecological interactions of complex microbial communities. These combined data sets offer a theoretical basis for accurate identification of target microorganisms, prediction of their nutritional requirements and optimization of cultivation conditions. For example, Jeremy Armetta and colleagues combined deep metagenomic sequencing with culturomics. They used a modified anaerobic medium and designed 50 different cultivation conditions. On this basis, they established a scalable method for targeted enrichment of gut microorganisms [144]. Culturomics, in turn, systematically builds diverse culture conditions and couples them with high-throughput isolation and rapid identification workflows. This strategy greatly improves access to low-abundance taxa and hard-to-culture microorganisms. It therefore provides important support for expanding culture collections and the diversity of cultivable microbial resources. Chou Wenhsuan developed a single-cell microliter-droplet culturomics system. This automated, high-throughput platform uses droplet microfluidic technology. Within one integrated workflow, it achieves single-cell separation, cultivation and screening of microorganisms [145]. Artificial intelligence and machine learning can analyze Raman spectra, microscopic images, and multi-omics data. These analytical tools predict the optimal culture medium composition and key environmental parameters. They also assist researchers in identifying target strains with specific metabolic functions or biosynthetic potential. Diao et al. developed an AI-driven Digital Colony Picker (DCP) technology [146]. Based on multimodal phenotypes, this system successfully enables the high-throughput sorting of strains. Looking forward, automated cultivation platforms will be increasingly integrated with microfluidic systems, single-cell sorting, and intelligent algorithms. Driven by this technological convergence, microbial isolation and cultivation are expected to move beyond traditional experience-driven models. Ultimately, the field will enter a new stage characterized by high throughput, precision, and intelligence.

In summary, microbial isolation and culture technology is transitioning from a discipline of random discovery to one of precision engineering. The field is currently undergoing multi-dimensional breakthroughs driven by the convergence of microfluidics, optics, data science, and microbial ecology. By moving toward fully automated, intelligent platforms that respect the ecological needs of single cells, we can transcend current limitations. These advancing tools promise to finally illuminate microbial “dark matter”, transforming undefined environmental resources into accessible biological assets for medicine, agriculture, and biotechnology.

## Figures and Tables

**Table 2:** Comparison of key characteristics and application scenarios of different microbial isolation and culture techniques.

Technology	Throughput	Resolution	Labeling Requirement	Application Scenario
Spread plate	Low	Single colony	None	Microbial isolation, purification, and enumeration; combinable with DNA sequencing for identification
Selective medium	Low	Specific community	None (Growth-based selection)	Targeted isolation of desired microbes from complex samples
Enrichment culture	Low	Specific community	None	Enrichment and preliminary screening of low-abundance or rare functional strains from environmental samples
Streaking and colony purification	Low	Single colony	None	Pure culture acquisition of most microorganisms; preliminary identification by colony morphology; routine strain purification
Membrane diffusion-based culture	Low	Specific community	None	Cultivation of uncultured/difficult-to-culture microbes (significantly improved); simulation of in situ conditions
Microfluidic technology	High Passive: ~10^6^ cells/min [60] Medium Active: ~10^4^ cells/min [73,80]	Single cell	None (Passive) Labelable (Active)	High-throughput screening; rare cell isolation; single-cell analysis
Optical tweezers	Medium HOT: 10^1^–10^2^ cells [97] Vortex: ~10 cells [101] LIFT: ~10^3^ cells [106]	Single cell	None	Precise single-cell manipulation; integration with complementary analytical techniques
FACS	High 10^5^–10^6^ cells/min [109]	Single cell	Requires fluorescence	Ultra-high-throughput sorting (e.g., mutant library screening); isolation of functional strains
RACS	Medium RAMS: 10^3^ cells/min [124] RTCS: <10 cells/min [129] RADS: 10^2^ cells/min [130] RACE: 10^2^–10^3^ cells/min [133]	Single cell	None	Label-free functional sorting (e.g., active degraders); real-time metabolic monitoring; combination with stable isotope probes
Reverse genomics screening	Low	Single cell	Requires (e.g., Probes)	Targeted isolation of known-genome uncultured microbes; specific metabolic bacteria

**Table 3:** Comparison of the advantages, disadvantages, recovered microorganisms and applicability of different microbial isolation and cultivation techniques.

Technology	Advantages	Disadvantages	Suitability Analysis: Anaerobic Microorganisms & Extremophiles	Recovered Microorganisms
Spread plate	Simple, low cost, standardized, by morphological differentiation	Biased toward fast growers, contamination susceptibility, limited access to uncultured taxa	Anaerobic systems (chambers, jars, inert gases, reducing agents)	Common fast-growing bacteria, fungi, conventional culturable strains
Selective medium	High specificity, targeted isolation, effective background suppression	Dependence on prior physiological knowledge, limited discovery of novel taxa	Anaerobic systems (chambers, jars, inert gases, reducing agents)	*Termitomyces* sp. [18], *Listeria monocytogenes* [20]
Enrichment culture	Amplification of low abundance populations, increased recovery diversity, partial niche simulation	Severe community bias, limited purity	Anaerobic systems (chambers, jars, inert gases, reducing agents)	*Achromobacter* sp. *BP3* [24], *Acidithiobacillus ferriphilus QBS 3* [31]
Streaking and colony purification	Broad applicability, pure culture acquisition, morphology-based screening	Low throughput, tiny or slow growing colonies easily overlooked, single colony mixed	Anaerobic systems (chambers, jars, inert gases, reducing agents)	*Alcaligenes faecalis*, *Stenotrophomonas* sp., *Ochrobactrum* sp. [32], *Candida boidinii* [33]
Membrane diffusion-based culture	In situ like conditions, improved recovery of uncultured microbes	Long culture cycle, low throughput, complex setup	Reduces oxygen exposure, maintains natural microenvironment, suitable for anaerobic or oligotrophic microbes	*Oceanisphaera* sp., *Pseudomonas* sp., *Bacillus* sp., *Shewanella* sp. [46], *DAMO archaea*, *DAMO bacteria*, *Anammox bacteria* [48]
Microfluidic technology	High throughput, single-cell resolution, precise manipulation	High cost and technical barrier, chip standards inconsistent	Closed microchambers and droplets minimize oxygen, precise parameter control for extremophiles	*Haloferax volcanii* [74]
Optical tweezers	High precision single-cell manipulation, non-contact operation, compatibility with FAC Sand RACS	Subjective manual selection, Low throughput, specialized instrumentation, technical demands	Reduces repeated transfer and oxygen damage	*Nitrospira* sp. [92]
FACS	Ultra-high throughput, multi-parameter single-cell analysis	Fluorescent labeling requirement, potential cell damage	Compatible with anaerobic encapsulation presorting, rapid screening of extremophile populations	*Lactobacillus paraplantarum SNUP7* [113], *ANME archaea* [119]
RACS	Label-free sorting, functional screening, real-time metabolic detection, isotope compatibility	Weak natural Raman signals, moderate throughput, technical complexity, specialized instrumentation	Closed system sorting, identifies metabolically active anaerobes and extremophiles without labeling	*Marine Group II archaea* [134]
Reverse genomics screening	Genome-guided targeting, access to uncultured taxa, high specificity	Depends on high-quality genome data, probe design dependence	Precisely matches anaerobic and extreme parameters, predicts cofactors and nutritional needs	*Nanopusillus acidilobi*, *Acidilobus* sp. *7A* [139], *TM7 bacteria* [140]

Note: Only representative microorganisms are listed for each technique.

## Data Availability

No new data were created or analyzed in this study. Data sharing is not applicable to this article.

## Abbreviations

The following abbreviations are used in this manuscript:

FACS	Fluorescence-activated cell sorting
RACS	Raman-activated cell sorting
iChip	Isolation chip
DEP	Dielectrophoresis
HOT	Holographic optical tweezers
Vortex	Vortex beam optical tweezers
LIFT	Laser-induced forward transfer
RAMS	Raman-activated microfluidic sorting
RTCS	Raman tweezers cell sorting
RADS	Raman-activated droplet sorting
RACE	Raman-activated cell ejection
Active	Active microfluidics
Passive	Passive microfluidics

## References

[1] Jiao J., Liu L., Hua Z., Fang B., Zhou E., Salam N., Hedlund B., Li W. (2021). Microbial dark matter coming to light: Challenges and opportunities. Natl. Sci. Rev..

[2] Zamkovaya T., Foster J., de Crécy-Lagard V., Conesa A. (2021). A network approach to elucidate and prioritize microbial dark matter in microbial communities. ISME J..

[3] Matthews A., Lima-Zaloumis J., Ii R.V.D., Boyer G., Trembath-Reichert E. (2023). Heterotrophic growth dominates in the most extremotolerant extremophile cultures. Astrobiology.

[4] Zhang X.-H., Ahmad W., Zhu X.-Y., Chen J., Austin B. (2021). Viable but nonculturable bacteria and their resuscitation: Implications for cultivating uncultured marine microorganisms. Mar. Life Sci. Technol..

[5] Pande S., Kost C. (2017). Bacterial unculturability and the formation of intercellular metabolic networks. Trends Microbiol..

[6] Yan C., Owen J.S., Seo E.-Y., Jung D., He S. (2023). Microbial interaction is among the key factors for isolation of previous uncultured microbes. J. Microbiol..

[7] Giovannoni S.J., Thrash J.C., Temperton B. (2014). Implications of streamlining theory for microbial ecology. ISME J..

[8] Gou Z., Li J., He F., Bamao Z., Li Z., Xu T. (2023). Screening of a high-yield strain of avermectin B1a by colony analysis in situ. Int. Microbiol..

[9] dos Santos J., Joao S., Martín J., Vicente F., Reyes F., Lage O. (2022). iChip-Inspired isolation, bioactivities and dereplication of Actinomycetota from portuguese beach sediments. Microorganisms.

[10] Vilela C., Villela H., Rachid C., do Carmo F., Vermelho A., Peixoto R. (2021). Exploring the diversity and biotechnological potential of cultured and uncultured coral-associated bacteria. Microorganisms.

[11] Ossai J., Khatabi B., Nybo S., Kharel M. (2022). Renewed interests in the discovery of bioactive actinomycete metabolites driven by emerging technologies. J. Appl. Microbiol..

[12] Ji M., Ma B., Dong J., Liu S., Shi Y., Bu M., Wang L., Liu L. (2025). Mining microbial dark matter: Advanced cultivation techniques for bioactive compound discovery. Pharmaceuticals.

[13] Chen C., Nace G., Irwin P. (2003). A 6 × 6 drop plate method for simultaneous colony counting and MPN enumeration of *Campylobacter jejuni*, *Listeria monocytogenes*, and *Escherichia coli*. J. Microbiol. Methods.

[14] Huang Z., Han N., Chang Y., Li H., Ding L., Tan Y., Bi Y., Yang R., Wu J. (2023). Optimization of oligotrophic culturomics for isolation of human gut microbiota. Acta Microbiol. Sin..

[15] Kyule D.N., Maingi J.M., Njeru E.M., Nyamache A.K. (2022). Molecular characterization and diversity of bacteria isolated from fish and fish products retailed in kenyan markets. Int. J. Food Sci..

[16] Xun L., Huang R., Li Q., Meng Q., Su R., Wu X., Zhang R., Li L., Gong X., Dong K. (2025). Specialized metabolites present in Camellia reticulata nectar inhibit the growth of nectar-inhabiting microorganisms. Front. Plant Sci..

[17] Teramura H., Yasuda E., Naisei Y. (2021). Impact of spreading time to recovery rate in suitability test of solid agar media. Biocontrol Sci..

[18] Thomas R. (1985). Selective medium for isolation of Termitomyces from termite nests. Trans. Br. Mycol. Soc..

[19] Strauss T., Botha A., Greyling D., Mostert T., du Preez P., Kock J. (2000). Development and testing of selective media for mucoralean fungi. South Afr. J. Sci..

[20] Alzorkey N., Sandine W. (1990). Highly selective medium for isolation of listeria-monocytogenes from food. Appl. Environ. Microbiol..

[21] Fernandes E., Keyser C., Rangel D., Foster R., Roberts D. (2010). CTC medium: A novel dodine-free selective medium for isolating entomopathogenic fungi, especially metarhizium acridum, from soil. Biol. Control.

[22] Roy D. (2001). Media for the isolation and enumeration of bifidobacteria in dairy products. Int. J. Food Microbiol..

[23] Kai J., Chunling C., Yu H. (2024). Effect of activation and enrichment on soil microbial community structure. J. Microbiol..

[24] Hong Q., Dong X., He L., Jiang X., Li S. (2009). Isolation of a biphenyl-degrading bacterium, Achromobacter sp BP3, and cloning of the bph gene cluster. Int. Biodeterior. Biodegrad..

[25] Biktasheva L., Rudakova M., Ziniukov R., Selivanovskaya S., Galitskaya P. (2023). Composition of prokaryotic communities in anaerobic enrichment cultures of crude oil from romashkino oilfield (Russia). Geomicrobiol. J..

[26] Amer A., Kim Y. (2023). Rapid enrichment of cupriavidus necator in mixed microbial cultures using autotrophic growth: Mixed microbial cultures for biodegradable polymer production using CO_2_ and organic wastes. Environ. Eng. Sci..

[27] Liu G., Shan Y., Liu R., Sun C. (2023). Insights into the bacterial and archaeal population dynamics in the deep-sea hydrothermal sediments exposed to lights with different wavelengths. Deep Sea Res. Part I Oceanogr. Res. Pap..

[28] Kato S., Takashino M., Igarashi K., Mochimaru H., Mayumi D., Tamaki H. (2020). An iron corrosion-assisted H_2_-supplying system: A culture method for methanogens and acetogens under low H_2_ pressures. Sci. Rep..

[29] Wang M., Zheng N., Li X., Zhao K., Xie B. (2023). Enrichment pretreatment expands the microbial diversity cultivated from marine sediments. Microorganisms.

[30] Mu D., Liang Q., Wang X., Lu D., Shi M., Chen G., Du Z. (2018). Metatranscriptomic and comparative genomic insights into resuscitation mechanisms during enrichment culturing. Microbiome.

[31] Liu R., Liu S., Bai X., Liu S., Liu Y. (2025). Biooxidation of arsenopyrite by Acidithiobacillus ferriphilus QBS 3 exhibits arsenic resistance under extremely acidic bioleaching conditions. Biology.

[32] Surkatti R., Al Disi Z.A., El-Naas M.H., Zouari N., Van Loosdrecht M.C.M., Onwusogh U. (2021). Isolation and identification of organics-degrading bacteria from gas-to-liquid process water. Front. Bioeng. Biotechnol..

[33] Mota M.N., Palma M., Sá-Correia I. (2024). *Candida boidinii* isolates from olive curation water: A promising platform for methanol-based biomanufacturing. AMB Express.

[34] Wagner A.O., Markt R., Mutschlechner M., Lackner N., Prem E.M., Praeg N., Illmer P. (2019). Medium preparation for the cultivation of microorganisms under strictly anaerobic/anoxic conditions. J. Vis. Exp..

[35] Gordon D.F., Stutman M., Loesche W.J. (1971). Improved isolation of anaerobic bacteria from the gingival crevice area of man. Appl. Microbiol..

[36] Schultz J., Modolon F., Peixoto R.S., Rosado A.S. (2023). Shedding light on the composition of extreme microbial dark matter: Alternative approaches for culturing extremophiles. Front. Microbiol..

[37] Deming J.W., Baross J.A. (1986). Solid medium for culturing black smoker bacteria at temperatures to 120 °C. Appl. Environ. Microbiol..

[38] Quehenberger J., Albersmeier A., Glatzel H., Hackl M., Kalinowski J., Spadiut O. (2019). A defined cultivation medium for sulfolobus acidocaldarius. J. Biotechnol..

[39] Kaeberlein T., Lewis K., Epstein S.S. (2002). Isolating "uncultivable" microorganisms in pure culture in a simulated natural environment. Science.

[40] Mazumder A., Dobyns B., Howard M., Beckingham B. (2022). Theoretical and experimental considerations for investigating multicomponent diffusion in hydrated, dense polymer membranes. Membranes.

[41] Usman H., Davidson S., Manimaran N., Nguyen J., Seth R., Beckman E., Niepa T. (2021). Design of a well-defined poly(dimethylsiloxane)-based microbial nanoculture system. Mater. Today Commun..

[42] Crump J., Richardson G. (1985). The suitability of a membrane-diffusion growth chamber for studying bacterial interaction. J. Appl. Bacteriol..

[43] Lüchtrath C., Lamping F., Hansen S., Finger M., Magnus J., Büchs J. (2024). Diffusion-driven fed-batch fermentation in perforated ring flasks. Biotechnol. Lett..

[44] Hoess A., Thormann A., Friedmann A., Aurich H., Heilmann A. (2010). Co-cultures of primary cells on self-supporting nanoporous alumina membranes. Adv. Eng. Mater..

[45] Bollmann A., Lewis K., Epstein S. (2007). Incubation of environmental samples in a diffusion chamber increases the diversity of recovered isolates. Appl. Environ. Microbiol..

[46] Polrot A., Kirby J., Olorunniji F., Birkett J., Sharples G. (2022). iChip increases the success of cultivation of TBT-resistant and TBT-degrading bacteria from estuarine sediment. World J. Microbiol. Biotechnol..

[47] Modolon F., Schultz J., Duarte G., Vilela C., Thomas T., Peixoto R. (2023). In situ devices can culture the microbial dark matter of corals. Iscience.

[48] Ding Z., Lu Y., Fu L., Ding J., Zeng R.J. (2017). Simultaneous enrichment of denitrifying anaerobic methane-oxidizing microorganisms and anammox bacteria in a hollow-fiber membrane biofilm reactor. Appl. Microbiol. Biotechnol..

[49] Berdy B., Spoering A., Ling L., Epstein S. (2017). In situ cultivation of previously uncultivable microorganisms using the ichip. Nat. Protoc..

[50] Steinert G., Whitfield S., Taylor M., Thoms C., Schupp P. (2014). Application of diffusion growth chambers for the cultivation of marine sponge-associated bacteria. Mar. Biotechnol..

[51] Hornung R., Grünberger A., Westerwalbesloh C., Kohlheyer D., Gompper G., Elgeti J. (2018). Quantitative modelling of nutrient-limited growth of bacterial colonies in microfluidic cultivation. J. R. Soc. Interface.

[52] Li M., Raza M., Song S., Hou L., Zhang Z., Gao M., Huang J., Liu F., Cai L. (2023). Application of culturomics in fungal isolation from mangrove sediments. Microbiome.

[53] Lodhi A., Zhang Y., Adil M., Deng Y. (2023). Design and application of a novel culturing chip (cChip) for culturing the uncultured aquatic microorganisms. Arch. Microbiol..

[54] Zhao J., Shakir Y., Deng Y., Zhang Y. (2023). Use of modified ichip for the cultivation of thermo-tolerant microorganisms from the hot spring. BMC Microbiol..

[55] Solano D., Camacho-Leon S. (2025). ASSURED assessment of droplet-based microfluidics: A benchmark for its future development. Microsyst. Technol..

[56] Shen Y., Yalikun Y., Tanaka Y. (2019). Recent advances in microfluidic cell sorting systems. Sens. Actuators B Chem..

[57] Zhang T., Di Carlo D., Lim C., Zhou T., Tian G., Tang T., Shen A., Li W., Li M., Yang Y. (2024). Passive microfluidic devices for cell separation. Biotechnol. Adv..

[58] Zhou T., Zhang T., Yalikun Y., Hosokawa Y., Cui Q., Tian G., Feng S., Li M. (2026). Particle separation by passive microfluidics and their downstream assays. Sens. Actuators B Chem..

[59] Lee M., Choi S., Park J. (2011). Inertial separation in a contraction-expansion array microchannel. J. Chromatogr. A.

[60] Condina M., Dilmetz B., Bazaz S., Meneses J., Warkiani M., Hoffmann P. (2019). Rapid separation and identification of beer spoilage bacteria by inertial microfluidics and MALDI-TOF mass spectrometry. Lab A Chip.

[61] Barbosa V., Cerqueira L., Miranda J., Azevedo N. (2025). Yeast enrichment using microfluidic deterministic lateral displacement (DLD). Microchem. J..

[62] Kang X., Cha H., Nguyen N., Li W., Klimenko A., Zhang J., Yuan D. (2025). Viscoelastic microfluidics: Fundamentals, technological development and applications. Trac Trends Anal. Chem..

[63] Liu P., Liu H., Yuan D., Jang D., Yan S., Li M. (2021). Separation and enrichment of yeast saccharomyces cerevisiae by shape using viscoelastic microfluidics. Anal. Chem..

[64] Zhang T., Cain A., Semenec L., Pereira J., Hosokawa Y., Yalikun Y., Li M. (2023). Bacteria separation and enrichment using viscoelastic flows in a straight microchannel. Sens. Actuators B Chem..

[65] Lim H., Kim J., Choo S., Lee C., Han B., Lim C., Nam J. (2023). Separation and washing of candida cells from white blood cells using viscoelastic microfluidics. Micromachines.

[66] Liu P., Liu H., Semenec L., Yuan D., Yan S., Cain A., Li M. (2022). Length-based separation of Bacillus subtilis bacterial populations by viscoelastic microfluidics. Microsyst. Nanoeng..

[67] Yao J., Zhao K., Lou J., Zhang K. (2024). Recent advances in dielectrophoretic manipulation and separation of microparticles and biological cells. Biosensors.

[68] Jubery T., Srivastava S., Dutta P. (2014). Dielectrophoretic separation of bioparticles in microdevices: A review. Electrophoresis.

[69] Moncada-Hernández H., Lapizco-Encinas B. (2010). Simultaneous concentration and separation of microorganisms: Insulator-based dielectrophoretic approach. Anal. Bioanal. Chem..

[70] Yu J., Kawahisa M., Kinoshita A., Zulmajdi A., Mori T. (2024). Approaches for attaining clean bacterial fractions from complex environmental samples. Front. Mar. Sci..

[71] Thomas D., Kinskie K., Brown K., Flanagan L., Davalos R., Hyler A. (2025). Dielectrophoretic microfluidic designs for precision cell enrichments and highly viable label-free bacteria recovery from blood. Micromachines.

[72] Ganesan S., Juliet A. (2023). Computational analysis on design and optimization of microfluidic channel for the separation of Staphylococcus aureus from blood using dielectrophoresis. J. Braz. Soc. Mech. Sci. Eng..

[73] Gao W., Zhang C., Cai Y., Su F., Han C., Yu D., Luo Y., Xing X. (2024). Dielectrophoretic cell sorting with high velocity enabled by two-layer sidewall microelectrodes extending along the entire channel. Sens. Actuators B Chem..

[74] Duarte P.A., Menze L., Shoute L., Zeng J., Savchenko O., Lyu J., Chen J. (2021). Highly efficient capture and quantification of the airborne fungal pathogen sclerotinia sclerotiorum employing a nanoelectrode-activated microwell array. ACS Omega.

[75] Mogi K., Shirataki C., Kihara K., Kuwahara H., Hongoh Y., Yamamoto T. (2016). Trapping and isolation of single prokaryotic cells in a micro-chamber array using dielectrophoresis. RSC Adv..

[76] Fan Y., Wang X., Ren J., Lin F., Wu J. (2022). Recent advances in acoustofluidic separation technology in biology. Microsyst. Nanoeng..

[77] Gao Y., Wu M., Lin Y., Xu J. (2020). Acoustic microfluidic separation techniques and bioapplications: A review. Micromachines.

[78] Ai Y., Sanders C., Marrone B. (2013). Separation of escherichia coli bacteria from peripheral blood mononuclear cells using standing surface acoustic waves. Anal. Chem..

[79] Lee S., Cha B., Yi H., Kim J., Jeon J., Park J. (2024). Acoustofluidic separation of bacteria from platelets using tilted-angle standing surface acoustic wave. Sens. Actuators B Chem..

[80] Ding X., Peng Z., Lin S., Geri M., Li S., Li P., Chen Y., Dao M., Suresh S., Huang T. (2014). Cell separation using tilted-angle standing surface acoustic waves. Proc. Natl. Acad. Sci. USA.

[81] Ang B., Jirapanjawat T., Tay K., Ashtiani D., Greening C., Tuck K., Neild A., Cadarso V. (2024). Rapid concentration and detection of bacteria in milk using a microfluidic surface acoustic wave activated nanosieve. Acs Sens..

[82] Park B.S., Kye H.G., Kim T.H., Lee J.M., Ahrberg C.D., Cho E.-M., Yang S.I., Chung B.G. (2019). Continuous separation of fungal spores in a microfluidic flow focusing device. Analyst.

[83] Paryab A., Saghatchi M., Zarin B., Behsam S., Abdollahi S., Khachatourian A., Toprak M., Amukarimi S., Qureshi A., Niazi J. (2024). Magnetic particles-integrated microfluidics: From physical mechanisms to biological applications. Rev. Chem. Eng..

[84] Ennen I., Hütten A. Magnetic nanoparticles meet microfluidics. Proceedings of the 7th NRW Nano-Conference.

[85] Inglis D., Riehn R., Austin R., Sturm J. (2004). Continuous microfluidic immunomagnetic cell separation. Appl. Phys. Lett..

[86] Jo Y., Shen F., Hahn Y., Park J., Park J. (2016). Magnetophoretic sorting of single cell-containing microdroplets. Micromachines.

[87] Thi Y., Hoang B., Thanh H., Nguyen T., Ngoc T., Thu H., Hoang N., Bui T., Duc T., Quang L. (2022). Design and numerical study on a microfluidic system for circulating tumor cells separation from whole blood using magnetophoresis and dielectrophoresis techniques. Biochem. Eng. J..

[88] Ashkin A., Dziedzic J. (1989). Internal cell manipulation using infrared-laser traps. Proc. Natl. Acad. Sci. USA.

[89] Zhang H., Liu K. (2008). Optical tweezers for single cells. J. R. Soc. Interface.

[90] Mitchell J., Weller R., Beconi M., Sell J., Holland J. (1993). A practical optical trap for manipulating and isolating bacteria from complex microbial communities. Microb. Ecol..

[91] Xu T., Li Y., Han X., Kan L., Ren J., Sun L., Diao Z., Ji Y., Zhu P., Xu J. (2022). Versatile, facile and low-cost single-cell isolation, culture and sequencing by optical tweezer-assisted pool-screening. Lab A Chip.

[92] Nowka B., Off S., Daims H., Spieck E. (2015). Improved isolation strategies allowed the phenotypic differentiation of two Nitrospira strains from widespread phylogenetic lineages. FEMS Microbiol. Ecol..

[93] Chen H., Cheng C. (2022). Holographic optical tweezers: Techniques and biomedical applications. Appl. Sci..

[94] Curtis J., Koss B., Grier D. (2002). Dynamic holographic optical tweezers. Opt. Commun..

[95] Lafong A., Hossack W., Arlt J., Nowakowski T., Read N. (2006). Time-multiplexed Laguerre-Gaussian holographic optical tweezers for biological applications. Opt. Express.

[96] Hörner F., Woerdemann M., Müller S., Maier B., Denz C. (2010). Full 3D translational and rotational optical control of multiple rod-shaped bacteria. J. Biophotonics.

[97] Werner M., Merenda F., Piguet J., Salathé R., Vogel H. (2011). Microfluidic array cytometer based on refractive optical tweezers for parallel trapping, imaging and sorting of individual cells. Lab A Chip.

[98] Gahagan K., Swartzlander G. (1999). Simultaneous trapping of low-index and high-index microparticles observed with an optical-vortex trap. J. Opt. Soc. Am. B Opt. Phys..

[99] He H., Friese M., Heckenberg N., Rubinszteindunlop H. (1995). Direct observation of transfer of angular-momentum to absorptive particles from a laser-beam with a phase singularity. Phys. Rev. Lett..

[100] Jesacher A., Furhpater S., Bernet S., Ritsch-Marte M. (2004). Size selective trapping with optical "cogwheel" tweezers. Opt. Express.

[101] Zhang P., Hernandez D., Cannan D., Hu Y., Fardad S., Huang S., Chen J., Christodoulides D., Chen Z. (2012). Trapping and rotating microparticles and bacteria with moire-based optical propelling beams. Biomed. Opt. Express.

[102] Deng Y., Renaud P., Guo Z., Huang Z., Chen Y. (2017). Single cell isolation process with laser induced forward transfer. J. Biol. Eng..

[103] Serra P., Piqué A. (2019). Laser-induced forward transfer: Fundamentals and applications. Adv. Mater. Technol..

[104] Cheptsov V., Zhigarkov V., Maximova I., Minaev N., Yusupov V. (2023). Laser-assisted bioprinting of microorganisms with hydrogel microdroplets: Peculiarities of Ascomycota and Basidiomycota yeast transfer. World J. Microbiol. Biotechnol..

[105] Liang P., Liu B., Wang Y., Liu K., Zhao Y., Huang W., Li B. (2022). Isolation and culture of single microbial cells by laser ejection sorting technology. Appl. Environ. Microbiol..

[106] Wang Y., Xue Y., Wang H., Qu Y., Zhang K., Shang L., Liang P., Chen F., Tang X., Luo W. (2025). Automated laser-assisted single-cell sorting for cell functional and rna sequencing. ACS Sens..

[107] Chen F., Liu K., Shang L., Wang Y., Tang X., Liang P., Li B. (2024). Precision isolation and cultivation of single cells by vortex and flat-top laser ejection. Front. Microbiol..

[108] Hulett H., Bonner W., Barrett J., Herzenbe L.A. (1969). Cell sorting-automated separation of mammalian cells as a function of intracellular fluorescence. Science.

[109] Ma F., Xie Y., Huang C., Feng Y., Yang G. (2014). An improved single cell ultrahigh throughput screening method based on in vitro compartmentalization. PLoS ONE.

[110] Oberpaul M., Brinkmann S., Marner M., Mihajlovic S., Leis B., Patras M.A., Hartwig C., Vilcinskas A., Hammann P.E., Schäberle T.F. (2021). Combination of high-throughput microfluidics and FACS technologies to leverage the numbers game in natural product discovery. Microb. Biotechnol..

[111] Cho S.H., Chen C.H., Tsai F.S., Godin J.M., Lo Y.H. (2010). Human mammalian cell sorting using a highly integrated micro-fabricated fluorescence-activated cell sorter (μFACS). Lab A Chip.

[112] Skrekas C., Ferreira R., David F. (2022). Fluorescence-activated cell sorting as a tool for recombinant strain screening. Methods in Molecular Biology.

[113] Ahn J., Moon S., Lee M., Kwon M., Pyo J., Lee S., Kim H. (2025). Engineering polyphosphate-accumulating probiotics for therapeutic applications in chronic kidney disease. Food Biosci..

[114] May G.E., McManus C.J. (2022). High-throughput quantitation of yeast uORF regulatory impacts using FACS-uORF. Methods in Molecular Biology.

[115] Wang H., Xu S., Cai B., Qiu W., Lu H., Tang Y. (2025). Highly efficient gene editing of Feline herpesvirus 1 using CRISPR/Cas9 combined with FACS. Front. Cell. Infect. Microbiol..

[116] Genolet O., Ravid Lustig L., Schulz E.G. (2022). Dissecting molecular phenotypes through FACS-based pooled CRISPR screens. Methods in Molecular Biology.

[117] Tan Y., Zhang X., Feng Y., Yang G.-Y. (2022). Directed evolution of glycosyltransferases by a single-cell ultrahigh-throughput FACS-based screening method. Methods in Molecular Biology.

[118] Sallet H., Calvo M., Titus M., Jacquemin N., Meibom K.L., Bernier-Latmani R. (2025). High-throughput cultivation and isolation of environmental anaerobes using selectively permeable hydrogel capsules. ISME Commun..

[119] Hatzenpichler R., Connon S.A., Goudeau D., Malmstrom R.R., Woyke T., Orphan V.J. (2016). Visualizing in situ translational activity for identifying and sorting slow-growing archaeal− bacterial consortia. Proc. Natl. Acad. Sci. USA.

[120] Puppels G., Demul F., Otto C., Greve J., Robertnicoud M., Arndtjovin D., Jovin T. (1990). Studying single living cells and chromosomes by confocal raman microspectroscopy. Nature.

[121] Song Y., Yin H., Huang W. (2016). Raman activated cell sorting. Curr. Opin. Chem. Biol..

[122] Yan S., Qiu J., Guo L., Li D., Xu D., Liu Q. (2021). Development overview of Raman-activated cell sorting devoted to bacterial detection at single-cell level. Appl. Microbiol. Biotechnol..

[123] Tang X., Wu Q., Shang L., Liu K., Ge Y., Liang P., Li B. (2024). Raman cell sorting for single-cell research. Front. Bioeng. Biotechnol..

[124] Lindley M., Gala de Pablo J., Peterson J., Isozaki A., Hiramatsu K., Goda K. (2022). High-throughput raman-activated cell sorting in the fingerprint region. Adv. Mater. Technol..

[125] Wang X., Wang S., Diao Z., Hou X., Gong Y., Sun Q., Zhang J., Ren L., Li Y., Ji Y. (2025). Label-free high-throughput live-cell sorting of genome-wide random mutagenesis libraries for metabolic traits by Raman flow cytometry. Proc. Natl. Acad. Sci. USA.

[126] Wang X., Ren L., Diao Z., He Y., Zhang J., Liu M., Li Y., Sun L., Chen R., Ji Y. (2023). Robust spontaneous raman flow cytometry for single-cell metabolic phenome profiling via pDEP-DLD-RFC. Adv. Sci..

[127] Lee K., Pereira F., Palatinszky M., Behrendt L., Alcolombri U., Berry D., Wagner M., Stocker R. (2021). Optofluidic Raman-activated cell sorting for targeted genome retrieval or cultivation of microbial cells with specific functions. Nat. Protoc..

[128] Fang T., Shang W., Liu C., Xu J., Zhao D., Liu Y., Ye A. (2019). Nondestructive identification and accurate isolation of single cells through a chip with raman optical tweezers. Anal. Chem..

[129] Lee K., Palatinszky M., Pereira F., Nguyen J., Fernandez V., Mueller A., Menolascina F., Daims H., Berry D., Wagner M. (2019). An automated Raman-based platform for the sorting of live cells by functional properties. Nat. Microbiol..

[130] Wang X., Ren L., Su Y., Ji Y., Liu Y., Li C., Li X., Zhang Y., Wang W., Hu Q. (2017). Raman-activated droplet sorting (rads) for label-free high-throughput screening of microalgal single-cells. Anal. Chem..

[131] Jing X., Gong Y., Xu T., Meng Y., Han X., Su X., Wang J., Ji Y., Li Y., Jia Z. (2021). One-cell metabolic phenotyping and sequencing of soil microbiome by Raman-activated gravity-driven encapsulation (RAGE). Msystems.

[132] Jing X., Gou H., Gong Y., Su X., Xu L., Ji Y., Song Y., Thompson I., Xu J., Huang W. (2018). Raman-activated cell sorting and metagenomic sequencing revealing carbon-fixing bacteria in the ocean. Environ. Microbiol..

[133] Zhang J., Lin H., Xu J., Zhang M., Ge X., Zhang C., Huang W., Cheng J. (2024). High-throughput single-cell sorting by stimulated Raman-activated cell ejection. Sci. Adv..

[134] Wang Y., Xu J., Cui D., Kong L., Chen S., Xie W., Zhang C. (2021). Classification and identification of archaea using single-cell raman ejection and artificial intelligence: Implications for investigating uncultivated microorganisms. Anal. Chem..

[135] Diao Z., Jing X., Hou X., Meng Y., Zhang J., Wang Y., Ji Y., Ge A., Wang X., Liang Y. (2024). Artificial intelligence-assisted automatic Raman-activated cell sorting (AI-RACS) system for mining specific functional microorganisms in the microbiome. Anal. Chem..

[136] Zhang Y., Chang K., Ogunlade B., Herndon L., Tadesse L., Kirane A., Dionne J. (2024). From genotype to phenotype: Raman spectroscopy and machine learning for label-free single-cell analysis. ACS Nano.

[137] Quince C., Walker A.W., Simpson J.T., Loman N.J., Segata N. (2017). Shotgun metagenomics, from sampling to analysis. Nat. Biotechnol..

[138] Salam N., Xian W.-D., Asem M.D., Xiao M., Li W.-J. (2021). From ecophysiology to cultivation methodology: Filling the knowledge gap between uncultured and cultured microbes. Mar. Life Sci. Technol..

[139] Wurch L., Giannone R.J., Belisle B.S., Swift C., Utturkar S., Hettich R.L., Reysenbach A.-L., Podar M. (2016). Genomics-informed isolation and characterization of a symbiotic nanoarchaeota system from a terrestrial geothermal environment. Nat. Commun..

[140] Liu S., Moon C.D., Zheng N., Huws S., Zhao S., Wang J. (2022). Opportunities and challenges of using metagenomic data to bring uncultured microbes into cultivation. Microbiome.

[141] Cross K., Campbell J., Balachandran M., Campbell A., Cooper S., Griffen A., Heaton M., Joshi S., Klingeman D., Leys E. (2019). Targeted isolation and cultivation of uncultivated bacteria by reverse genomics. Nat. Biotechnol..

[142] Li J., Zhang D., Luo C., Li B., Zhang G. (2023). In situ discrimination and cultivation of active degraders in soils by genome-directed cultivation assisted by sip-raman-activated cell sorting. Environ. Sci. Technol..

[143] Liu H., Xue R., Wang Y., Stirling E., Ye S., Xu J., Ma B. (2021). FACS-iChip: A high-efficiency iChip system for microbial ‘dark matter’ mining. Mar. Life Sci. Technol..

[144] Armetta J., Li S.S., Vaaben T.H., Vazquez-Uribe R., Sommer M.O.A. (2025). Metagenome-guided culturomics for the targeted enrichment of gut microbes. Nat. Commun..

[145] Chou W., Guo X., Wang L., Xing X.-H., Wang Y., Zhang C. (2025). Automated and high-throughput microbial monoclonal cultivation and picking using the single-cell microliter-droplet culture omics system. J. Vis. Exp..

[146] Diao Z., Peng Q., Luo S., Kan L., Ge A., Gao W., Li R., Bao W., Wang X., Ji Y. (2025). AI-powered high-throughput digital colony picker platform for sorting microbial strains by multi-modal phenotypes. Nat. Commun..

